# Coma-corrected rapid single-particle cryo-EM data collection on the CRYO ARM 300

**DOI:** 10.1107/S2059798321002151

**Published:** 2021-04-14

**Authors:** Rouslan G. Efremov, Annelore Stroobants

**Affiliations:** a Center for Structural Biology, Vlaams Instituut voor Biotechnologie, Pleinlaan 2, 1050 Brussels, Belgium; b Structural Biology Brussels, Vrije Universiteit Brussel, Pleinlaan 2, 1050 Brussels, Belgium

**Keywords:** single-particle cryo-EM, high throughput, high-resolution 3D reconstruction

## Abstract

Single-particle cryogenic electron microscopy has recently become a major method for determining the structures of proteins and protein complexes. This has markedly increased the demand for throughput of high-resolution electron microscopes, which are required to produce high-resolution images at high rates. An increase in data-collection throughput can be achieved by using large beam-image shifts combined with off-axis coma correction, enabling the acquisition of multiple images from a large area of the EM grid without moving the microscope stage. Here, the optical properties of the JEOL CRYO ARM 300 electron microscope equipped with a K3 camera were characterized under off-axis illumination conditions. It is shown that efficient coma correction can be achieved for beam-image shifts with an amplitude of at least 10 µm, enabling a routine throughput for data collection of between 6000 and 9000 images per day. Use of the benchmark for the rapid data-collection procedure (with beam-image shifts of up to 7 µm) on apoferritin resulted in a reconstruction at a resolution of 1.7 Å. This demonstrates that the rapid automated acquisition of high-resolution micrographs is possible using a CRYO ARM 300.

## Abstract 2

Coma-corrected single-particle data collection on a JEOL CRYO ARM 300 is described. It is shown that data collection with a throughput exceeding 6000 images per day and an image shift of up to 7 µm produces a reconstruction of apoferritin to a resolution of 1.7 Å.

## 1. Introduction   

Density maps of proteins can be reconstructed at atomic resolution using single-particle cryogenic electron microscopy (cryo-EM; Nakane *et al.*, 2020 ▸; Yip *et al.*, 2020 ▸). Although intense development of the method continues, single-particle cryo-EM has already revolutionized structural biology over the course of the last six years. Today, obtaining 3D reconstructions of proteins and protein complexes at near-atomic resolution (better than 4 Å) has become routine. The rapid progress in single-particle cryo-EM has been enabled by the introduction and perfection of direct electron detectors (Ruskin *et al.*, 2013 ▸) and by improvements in image-processing software (Scheres, 2012 ▸; Grant *et al.*, 2018 ▸; Punjani *et al.*, 2017 ▸). High-resolution imaging imposes stringent constraints on electron microscopes, as images with minimal subatomic drift and minimal aberrations are required to preserve information at atomic resolution (∼1 Å). The development of dedicated high-resolution, stable and fully automated electron microscopes, which are equipped with energy filters and produce highly coherent parallel illumination over large illumination areas, has also played a critical role in the success of the method.

The collection of a single-particle data set involves the acquisition of thousands of images from a vitrified protein solution on a cryo-EM grid, and this process is enabled by highly reliable automation of the data-collection process. Multiple software packages facilitating automated data collection, including, but not limited to, *Leginon* (Suloway *et al.*, 2005 ▸), *SerialEM* (Mastronarde, 2005 ▸) and *EPU* (Thermo Fisher Scientific), have been developed. These software packages allow their users to visualize the EM grid at different magnifications. Initially, an atlas of the complete grid is recorded. This is used to select good grid squares before the positions for image recording are chosen. Automated data collection occurs next, executing a protocol with defined data-acquisition parameters.

Modern crystallographic beamlines on synchrotrons have a very high throughput: the collection of a diffraction data set takes only a few minutes (Svensson *et al.*, 2015 ▸), whereas the collection of single-particle cryo-EM data sets often takes days. The lower single-particle cryo-EM data-collection throughput is because of the necessity of collecting thousands of images at spatially separated positions on a cryo-EM grid. This is achieved by displacing the sample stage with sub­micrometre accuracy, followed by a delay to stabilize stage drift (Suloway *et al.*, 2005 ▸).

Recently, a new data-collection strategy has been introduced. It uses a beam-image shift with an amplitude ranging from few micrometres to tens of micrometres to collect single-particle images from a large area of the EM grid without displacing the sample stage (Wu *et al.*, 2019 ▸; Cheng *et al.*, 2018 ▸). This approach significantly increases the data-collection throughput. The beam-image shift, however, induces coma and astigmatism (Wu *et al.*, 2019 ▸; Cheng *et al.*, 2018 ▸; Glaeser *et al.*, 2011 ▸). Coma results in a phase error that grows at third and higher powers of spatial frequency (Glaeser *et al.*, 2011 ▸); therefore, it primarily influences high-resolution shells (4 Å and higher) of the 3D reconstruction and becomes increasingly strong as one approaches atomic resolution (Zivanov *et al.*, 2020 ▸). Beam-image shift-induced aberrations need to be optically corrected in order to maintain the high quality of the homogeneous single-particle data over the entire area used for data collection. The accuracy of the beam-image shift-induced coma correction, in combination with the absence of image shift-dependent higher-order aberrations (which cannot be corrected for), defines the success of this strategy for single-particle data collection.

The JEOL CRYO ARM 300 is a new-generation 300 kV electron cryogenic microscope released in 2018. It is equipped with a cold field emission gun (cFEG), an in-column Ω energy filter, a four-lens condenser and an autoloader for 12 samples. It has been shown that the CRYO ARM 300 enables 3D reconstruction to a resolution of 1.54 Å for apoferritin (Kato *et al.*, 2019 ▸). The performance of the CRYO ARM 300 has been characterized in multiuser environment settings (Fislage *et al.*, 2020 ▸; Hamaguchi *et al.*, 2019 ▸), but not for high-throughput data collection using coma-corrected beam-image shift.

Here, we describe procedures for rapid data collection using a coma-corrected beam-image shift on a CRYO ARM 300 using *SerialEM*. We characterized the optical properties of our CRYO ARM 300 microscope with applied beam-image shifts and assessed the efficiency of image shift-induced coma and astigmatism correction. Furthermore, we benchmarked this data-collection strategy with image shifts of up to 7 µm on mouse heavy-chain apoferritin and achieved a reconstruction at a resolution of 1.7 Å. Altogether, our results demonstrate the suitability of a CRYO ARM 300 for rapid and high-resolution single-particle data collection. They also indicate the limiting factors which need to be eliminated to improve both the throughput of data collection and the resolution of single-particle 3D reconstructions.

## 2. Materials and methods   

### 2.1. Purification of apoferritin and preparation of cryo-EM grids   

The plasmid with mouse heavy-chain apoferritin was a gift from Dr H. Yanagisawa (University of Tokyo). Apoferritin was expressed and purified following a procedure described previously (Fislage *et al.*, 2020 ▸), with the only modification being that the reducing agent tris(2-carboxyethyl)phosphine (TCEP) was added at a concentration of 1 m*M* to the purification buffers to prevent protein aggregation during the preparation of the cryo-EM sample (Wu *et al.*, 2020 ▸).

Cryo-EM grids were prepared by applying 3 µl purified apoferritin at a concentration of approximately 3.6 mg ml^−1^ to 200-mesh holey Quantifoil R 1.2/1.3 carbon grids that had been glow-discharged for 1 min in an ELMO glow-discharge system (Cordouan). The grids were blotted with Whatman Grade 1 paper for 5 s with offset 0 at 25°C and 98% humidity, after which they were immediately plunged into liquid ethane at −170°C using a Cryoplunge 3 (Gatan).

### 2.2. Measurements of beam-image shift-induced coma and astigmatism   

Measurements of coma and astigmatism, as well as the acquisition of an apoferritin data set, were performed on a CRYO ARM 300 with spot size 6, using alpha angle 1 with a condenser aperture of 150 µm and the objective aperture retracted. The energy-filter slit was centered on the zero-loss peak, and the slit width was set to 20 eV. Images were recorded on a K3 detector (Gatan) operating in correlated double-sampling (CDS) mode, with an exposure of 3.37 s partitioned into 59 frames. A dose of approximately 1 e^−^ Å^−2^ was exposed per frame, at a nominal magnification of 60 000 and with a corresponding magnified pixel size of 0.75 Å.

Beam-image shift-induced coma was measured using a grating grid (Electron Microscopy Sciences) with latex spheres. Beam-image shifts with a radius of between 2 and 10 µm, in 2 µm steps, were applied along 16 equi-angularly spaced directions (angular step 22.5°) and the beam tilt was measured using the *SerialEM*3.8.0 command FixComaByCTF. After coma correction, an image was recorded and the astigmatism was measured by fitting the power spectrum using *CTFFIND*4 (Rohou & Grigorieff, 2015 ▸) after frame alignment in *MotionCor*2 (Zheng *et al.*, 2017 ▸). The astigmatism angle was defined as per the study conducted by Mindell & Grigorieff (2003 ▸) and the amplitude was calculated as the difference between the maximum and minimum values of defocus. The measurements were performed automatically using a home-written script both with and without applying coma and astigmatism correction. For the measurements, the grid *z*-height was offset from the standard focus by a distance of between 1 and 2 µm, and the current in the objective lens was close to the standard focus, within an equivalent defocus of 1 µm.

### 2.3. Measurements of residual beam-image shift   

Residual drift, arising from the potential relaxation of the deflectors after setting the beam-image shift with the corresponding adjustment of the beam tilt and astigmatism, was measured in *SerialEM*3.8.0. For these measurements, additional delays incorporated in *SerialEM* were zeroed by setting the image-shift delay factor and the property AstigBTBacklashDelay to 0.

A script implementing beam-image shift from the neutral position to a position with a given amplitude of image shift, and at equally spaced angles, was applied. This was followed by immediate image recording of the grating grid on the K3 detector operating in CDS mode, with a total exposure of 4.98 s partitioned over 87 frames and a flux of approximately 1 e^−^ Å^−2^ per frame. The time for each cycle of script execution was verified to ensure the absence of any additional delays, and it was found to be equal to the sum of the time required for both beam-image tilt–astigmatism application and image recording to within 0.4–0.6 s. The recorded frames were aligned in *MotionCor*2, and the resulting trajectory was used to analyze the residual image shift.

### 2.4. Pivot-point alignment   

An accurate alignment of compensation for beam shift is critical for minimizing beam-shift-induced coma. To align the pivot points, the grid was brought to standard focus height and the beam diameter was carefully adjusted by minimizing the beam-diameter variation during objective lens wobbling, ensuring that the beam was parallel. The objective lens was defocused to between 40 and 100 µm. Next, the condenser aperture was removed and the beam shift was wobbled using *SerialEM*, with the largest amplitude maintaining the fluorescent screen illuminated at the extreme beam positions. The beam-shift compensator was adjusted to minimize the movement of a feature on the grid along the wobbling direction.

Following the adjustment of pivot points, refinement of the beam shift against image shift at large image-shift amplitudes (up to 10 µm) was performed in *SerialEM* to ensure precise beam centering at large beam-image shifts using the *SerialEM* Refine Beam Shift Cal command.

### 2.5. Single-particle data collection and image processing   

A single-particle cryo-EM data set for apoferritin was collected using the CRYO ARM 300 electron microscope at a nominal magnification of 60 000. The microscope illumination conditions were set to spot size 6 and alpha 1. A condenser aperture of 150 µm was used to produce an illumination area of approximately 1.5 µm in diameter, and the objective aperture was retracted. The energy-filter slit was centered on the zero-loss peak, with the slit width set to 20 eV. Images were recorded on a K3 detector (Gatan) operating in CDS mode, with an exposure time of 3.37 s partitioned onto 59 frames. The corresponding electron dose was 1 e^−^ Å^−2^ per frame, the corresponding flux on the detector was 10 e^−^ per pixel per second and the calibrated magnified pixel size was 0.753 Å.

Before data collection, calibration of coma and astigmatism matrices versus image shift was performed in *SerialEM*3.8.0 using a grating grid. Coma-free alignment was performed before launching the data collection. Data were collected using *SerialEM*3.8.0 from a pattern of 5 × 5 holes on a Quantifoil R 1.2/1.3 grid, recording one image in the center of each hole. The holes were sampled along a serpentine-shaped path starting from a corner of the pattern. Focusing was performed once per stage position on the carbon area next to the central hole. Focusing within a limit of ±5 µm from the standard focus was performed by adjusting the objective lens current. For larger defoci, the *z*-height of the stage was adjusted before the objective lens current was used to focus. A data set of 3125 images was collected from 125 stage positions. After frame alignment in *MotionCor*2 and determination of CTF parameters in *CTFFIND*4 (Rohou & Grigorieff, 2015 ▸), the images were assessed with the help of *BXEMDALYSER* (a home-written program that allows the user-friendly selection of micrographs based on their visual appearances, parameters of frame alignment and CTF fits; unpublished work). Images with thick or crystalline ice and those containing multiple layers of apoferritin particles were discarded.

From the remaining 2639 micrographs, a total of 1 514 772 particles picked in *crYOLO*1.5.6 (Wagner *et al.*, 2019 ▸) were extracted in a box of 140 pixels binned twice. The particles were subjected to a 2D classification into 150 classes performed with the ‘ignore until first CTF peak’ option selected in *RELION*3.1 (Zivanov *et al.*, 2020 ▸). Further processing was performed in *RELION*3.1. After two rounds of 2D classification, 1 326 632 particles were selected for further processing. Unmasked initial 3D auto-refinement, performed on particles extracted into a box size of 280 pixels and against the initial model filtered to 40 Å, converged to a resolution of 2.6 Å. Refinement of beam tilt indicated an *X*/*Y* tilt of −0.25/−0.65 mrad. After tilt correction and per-particle defocus refinement, the resolution of 3D reconstruction improved to 2.1 Å.

Following Bayesian polishing and another round of CTF refinement, including trefoil and fourth-order aberration, anisotropic magnification and retention of particles with defocus below 1.2 µm to avoid CTF aliasing (Penczek *et al.*, 2014 ▸), a total of 702 667 particles were retained and the resolution further improved to 1.79 Å. Next, individual optics groups were created for data collected from each stage position and the beam tilt was refined for each optics group. This further improved the resolution to 1.71 Å. The Rosenthal–Henderson *B* factor was calculated using the bfactor_plot.py script (Zivanov *et al.*, 2018 ▸). Real-space refinement of the apoferritin model (PDB entry 3wnw; R. Zarivach & L. Lewin, unpublished work) against the final map was performed in *Phenix*1.14 (Afonine *et al.*, 2013 ▸), and the map–model FSC was calculated using the *phenix.mtriage* routine (Afonine *et al.*, 2018 ▸).

To evaluate the quality of single-particle data recorded at individual positions in the 5 × 5 pattern, the polished particles were split into optics groups containing images with identical beam-image shifts. Beam tilt and higher-order aberrations were refined for each optics group. Next, 3D auto-refinement with local angular sampling, starting from 1.8°, was calculated for each optics group separately. This was followed by calculation of the Rosenthal–Henderson *B* factor for each subset.

## 3. Results   

### 3.1. How fast can single-particle data be collected?   

We first asked the question: how fast can single-particle data be collected? In the process of single-particle data collection, multiple operations are executed on the microscope at different frequencies. To optimize throughput, it is helpful to have a model that can be used to evaluate the dependence of the throughput on the duration of the individual steps.

**Figure 1:**
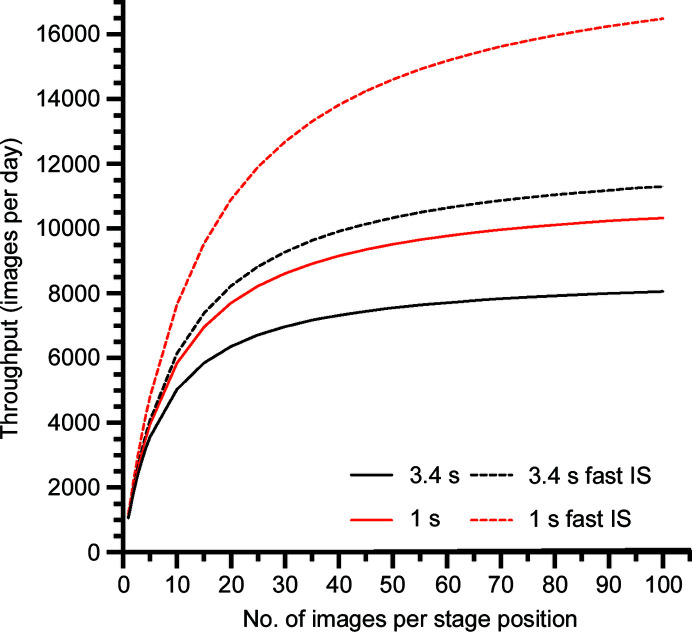
Daily throughput of micrograph acquisition as a function of number of images recorded per stage position. Curves are shown for exposure times of 3.4 and 1 s for the currently set up data-acquisition procedure (solid lines). The corresponding saturation throughputs are 8600 and 11 300 images per day, respectively. Dashed lines show hypothetical throughputs, assuming that *t*_BS_ is reduced from 4 to 1 s. The corresponding saturation throughputs are 12 500 and 18 900 images per day, respectively.

Table 1 ▸ summarizes the individual steps involved in the data-collection process, as well as the characteristic times per step measured for the procedures as established using our microscope. The throughput, *N*, is defined as the number of micrographs collected per 24 h and can be estimated using 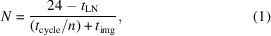
where *n* is the number of micrographs recorded per stage position, *t*_cycle_ = *t*_stage_ + *t*_focus_ + *t*_beam_ + *t*_ice_ is the cumulative time of operations performed only once per stage position, and *t*_img_ = *t*_BS_ + *t*_stab_ + *t*_rec_ + *t*_SerialEM_ is the cumulative time for collecting one micrograph (units of time should be measured in hours for this equation). The corresponding values of *t*_cycle_ and *t*_img_ for the data-collection routine set up on our microscope are 66 and 7.5–9.8 s, respectively. The dependence of the throughput on the number of micrographs recorded per stage position, for the combination of parameters discussed below, is shown in Fig. 1 ▸. The throughput increases rapidly as the number of images acquired per stage position increases, and for values of *n* such that *n* ≫ *t*_cycle_/*t*_img_, the throughput is defined by *t*_img_ only. The rate of increase in throughput depends on the ratio *t*_cycle_/*t*_img_, and it reaches half-maximum when *n* is equal to *t*_cycle_/*t*_img_ (between 7 and 8 for the timings listed in Table 1 ▸).

When a few tens, or more, of images are recorded per stage position, *t*_img_ becomes the main determinant of data-collection throughput (equation 1 and Fig. 1 ▸). Therefore, we looked closely at the duration of the individual steps contributing to *t*_img_. The time required for image recording (*t*_rec_), the main contributor to *t*_img_, consists of the actual exposure time plus the dead time of the camera. For the flux recommended for the K3 camera (between 8 and 40 e^−^ per pixel per second), a pixel size of 0.75 Å (corresponding to a magnification of 60 000 used for routine data collection on our microscope) and a cumulative electron dose of ∼60 e^−^ Å^−2^, the exposure times are between 2.15 and 3.38 s. An additional overhead of approximately 2 s, which does not depend on the exposure settings, is associated with the recording of an image (Supplementary Table S1). Reducing the total exposure to 30 e^−^ Å^−2^, which is common for single-particle data-collection procedures, can further reduce the image-recording time to 3.3 s.

After eliminating all of the delays encoded in *SerialEM* during the process of setting the beam-image shift and adjusting the beam tilt and astigmatism, *t*_BS_ was measured to be 4 s. This time is surprisingly long, and considerably impacts the saturation throughput, creating an unexpected bottleneck in the data-collection throughput. We experimentally verified whether the stabilization delay (*t*_stab_), the time after application of the beam-image shift and before acquisition of the EM image, is needed to ensure that potential relaxation of deflectors does not cause residual image drift. To do this, a movie was recorded on a grating immediately after shifting the beam, adjusting the beam tilt and astigmatism for various directions and the amplitudes of the beam-image shift. If a significant residual relaxation of image shift occurred, an apparent residual drift should be visible on the trajectories of the aligned frames. Supplementary Fig. S1 shows that after an image shift of 10 µm, a linear drift is observed over a distance of between 0.2 and 1.5 Å. Because of its variable amplitude and the absence of correlation between the direction of image shift and the direction of apparent drift, it is most likely caused by a charging effect rather than by relaxation of the deflectors. Moreover, owing to the high rate of fractionation, the associated shifts per frame were always below 0.4 Å, which does not influence the image quality at resolutions lower than 1.2 Å (Fislage *et al.*, 2020 ▸). Thus, we can conclude that deflector-stabilization delay (*t*_stab_) is not required during data collection.

In conclusion, given the parameters established for data-collection procedures on our CRYO ARM 300, throughput limits of between 8600 and 11 300 images per day can be expected. In practice, for a limited number of images recorded per stage position, throughputs of between 6000 and 9000 images per day can be reached. It is important to note, however, that reducing *t*_BS_ from 4 to 1 s would further accelerate the collection rate to the range of between 9000 and 17 000 images per day (Fig. 1 ▸).

### 3.2. Beam-image shift-induced coma and its correction   

Moving the beam away from the optical axis induces coma and astigmatism, the magnitudes of which depend on the microscope alignment and the properties of the objective lens (Glaeser *et al.*, 2011 ▸; Cheng *et al.*, 2018 ▸). Both aberrations can be efficiently corrected for: changing the beam tilt corrects for coma and adjusting the objective lens stigmators can compensate for astigmatism (Wu *et al.*, 2019 ▸).

The range of accessible beam shifts on the CRYO ARM 300 has limits that depend on the illumination angle alpha. Thus, at alpha 1, which is typically used for data collection with a K3 detector, the beam-shift amplitude is limited to approximately 15 µm. At alpha 2, however, the limit extends to approximately 40 µm. Therefore, alpha 2 may need to be used for beam-image shifts with amplitudes above 15 µm.

**Figure 2:**
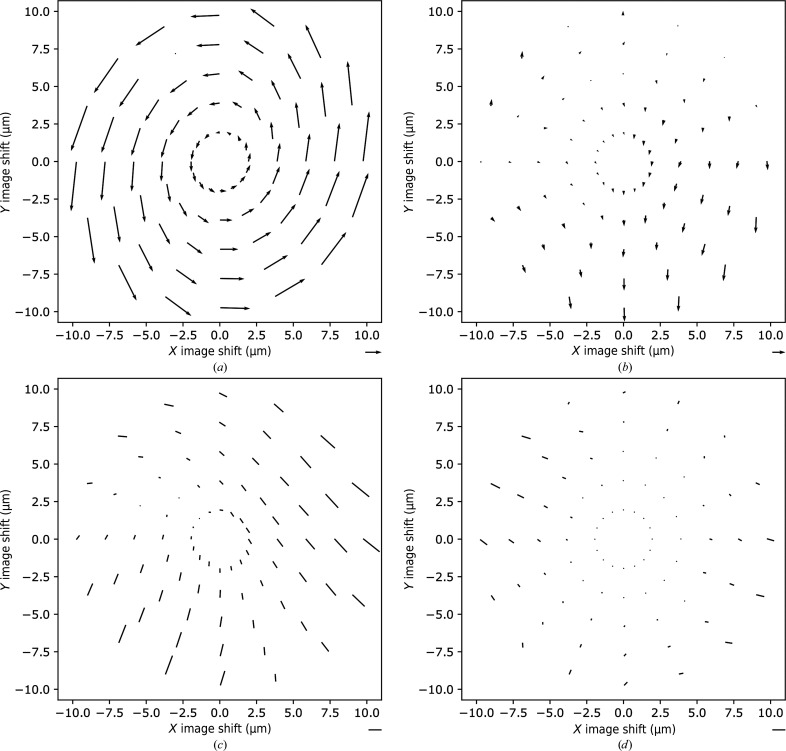
Vector plots of beam-image shift-induced coma and astigmatism. Vectors indicate the amplitude and direction of the beam-image shift-induced beam tilt (*a*) before and (*b*) after correction. The scale bar for (*a*) is 1 mrad and that for (*b*) is 0.2 mrad. Lines show the amplitude and direction of astigmatism, as defined by Mindell & Grigorieff (2003 ▸), (*c*) before and (*d*) after correction. The scale bars in both panels are 2000 Å.

Inaccuracy in the alignment of pivot points contributes to beam-induced coma. We have noticed that the standard pivot-point alignment procedure, which utilizes the diffraction mode, was not accurate enough and had to be improved. Following the suggestion of Dr Sohei Motoki (JEOL), we modified the alignment procedure to adjust image-shift compensation in imaging mode. This modification is described in more detail in Section 2. To evaluate the optical properties of the microscope, we measured the beam-image shift-induced coma and astigmatism for a range of image shifts up to 10 µm (Fig. 2 ▸ *a*).

The beam-image shift-induced coma was nearly perpendicular to the direction of the image shift, as existing theory had led us to expect (Glaeser *et al.*, 2011 ▸), and had an elliptical dependence on the amplitude of the image-shift direction (Supplementary Fig. S2*a*). Coma amplitude increases linearly as the amplitude of the image shift increases, with slopes of 0.19 and 0.31 mrad µm^−1^ for the minor and major elliptical axis directions, respectively (Supplementary Fig. S2*c*).

The coma remaining after applying beam-tilt correction (using a linear matrix dependent on image shifts), as implemented in *SerialEM*3.8.0, is shown in Fig. 2 ▸(*b*) and Supplementary Fig. S2(*c*). The fivefold difference in the length of the scale bar between Figs. 2 ▸(*a*) and 2 ▸(*b*) should be noted. For most image-shift positions, beam-tilt correction reduces the coma to below 0.1 mrad. However, a certain level of residual coma remains for some directions, revealing an asymmetry in the beam-image shift-induced coma. The center of the residual coma pattern is shifted towards the top left corner of the sampled beam-image shift space, and the residual coma reaches up to 0.25 mrad at the largest beam-image shift. However, these values of residual coma should still allow single-particle reconstruction to a resolution above 2.4 Å (Glaeser *et al.*, 2011 ▸).

**Figure 3:**
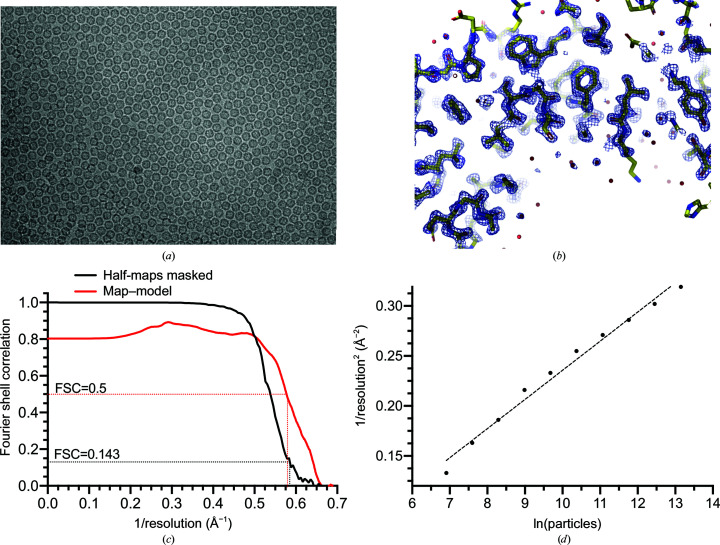
Reconstruction of apoferritin from a data set collected from a 5 × 5 pattern on a Quantifoil R 1.2/1.3 grid. (*a*) Example micrograph of apoferritin. (*b*) Density map at a resolution of 1.7 Å. (*c*) Fourier shell correlation curve for the masked reconstruction and between the refined model and the map. (*d*) Rosenthal–Henderson *B*-factor plot fitted with a *B* factor of 68 Å^2^.

The pattern of beam-image shift-induced astigmatism is significantly more asymmetric than that of coma (Fig. 3 ▸ *c*, Supplementary Fig. S3*a*) The amplitude of astigmatism reaches up to 3500 Å for an image shift of 10 µm. The center of the astigmatism pattern appears to be located in roughly the same position as the center of the residual coma pattern (Fig. 3 ▸ *b*), suggesting that the asymmetric behaviors of both coma and astigmatism may share an origin. In spite of its asymmetric character, astigmatism can be efficiently reduced by adjusting the objective lens stigmators using a simple linear transformation of image-shift values, as was implemented in *SerialEM*3.8.0 (Fig. 3 ▸ *d*, Supplementary Fig. S3*b*). Upon correction, the astigmatism is reduced to below 300 Å for image shifts up to 4 µm and to below 1600 Å for a beam-image shift with an amplitude of 10 µm (Supplementary Fig. S3*b*). The magnitude of residual astigmatism is significantly lower than the amplitude of defocus (0.5 µm and higher); therefore, if determined accurately by defocus-fitting programs, the residual astigmatism should not interfere with downstream image processing and CTF correction. More accurate astigmatism correction can be achieved by fitting beam-image shift-induced astigmatism with a more sophisticated empirical function, as, for example, realized by Wu *et al.* (2019 ▸).

### 3.3. Benchmark with apoferritin   

To benchmark rapid data collection with significant image shifts, we collected a data set for mouse heavy-chain apo­ferritin. The density of apoferritin particles on the cryo-EM grid improved compared with our previously reported benchmark (Fislage *et al.*, 2020 ▸), resulting in a tightly packed layer of particles (Fig. 3 ▸ *a*). This was achieved by adding a reducing agent to the purification buffers, similar to the work of Wu *et al.* (2020 ▸). Further details can be found in Section 2. It is likely that under oxidizing conditions a surface-exposed cysteine (Cys102) cross-links apoferritin molecules with cysteine bridges as the protein concentrates at the air–water interface during the preparation of the cryo-EM sample (Armstrong *et al.*, 2019 ▸). This causes protein aggregation and a reduction of particle density in the holes of the EM grid.

We estimated that the objective aperture limits the resolution to the range between 1.5 and 2.0 Å. Therefore, the objective aperture was retracted for this benchmark. 25 images were recorded per stage position from a 5 × 5 array of holes on a Quantifoil R 1.2/1.3 grid. One image was recorded in the center of each hole of the array. With these settings, the largest image shifts were 7 µm at the corners of the square pattern. The data set was collected with the beam size expanded to 1.5 µm to cover the carbon area around the hole, which aimed to reduce the charging effect that we regularly observed with apoferritin samples. This is different from regular conditions, in which a beam with a diameter of 1 µm is used, collecting three images per 1.2 µm hole and five per 2 µm hole.

A data set of 3125 images acquired from 125 stage positions was collected in 12 h (the refilling of liquid-nitrogen dewars occurred during this time). This corresponds to a data-acquisition rate of 6250 images per day, which is within a 10% margin of the expected rate of 6717 (Fig. 1 ▸). This data-collection rate is 2.6 times higher than the previous fastest data-collection rate that we have reported, which was achieved with a K2 camera and was collected by recording five images per stage position (Fislage *et al.*, 2020 ▸).

A 3D reconstruction of apoferritin was calculated to a resolution of 1.71 Å from 702 667 particles imaged with a defocus below 1.2 µm (Table 2 ▸, Fig. 3 ▸ *b*). A *B* factor of 68 Å^2^ was also determined from the *B*-factor plot (Rosenthal & Henderson, 2003 ▸; Fig. 3 ▸ *d*). The 3D map displayed the features expected at this resolution (Fig. 3 ▸ *b*), including the holes in the imidazole rings of histidines and in the five-membered pyrrole rings of tryptophan side chains. Many C atoms displayed separated density blobs at higher contour levels. Refinement of optical aberrations in accordance with the work of Zivanov *et al.* (2020 ▸) revealed a surprisingly high average beam tilt of 0.9 mrad in the data set, which was present due to the instability of the beam tilt at the beginning of data collection. This beam-tilt instability on our microscope is a recurrent problem which requires further examination.

To obtain the reconstruction, processing with a single optics group was performed; that is, neither beam tilt nor aberration correction were refined separately for images recorded with a shifted beam. Before the last reconstruction, individual optics groups were created for groups of images recorded from the same stage position. The beam tilt for each group was refined independently to account for instabilities in beam tilt. The overall quality of these data is comparable to the reconstruction obtained from data recorded close to the optical axis and at higher magnification on a K2 detector (Fislage *et al.*, 2020 ▸), indicating that larger image shifts do not cause the quality of the single-particle data to deteriorate.

To further understand whether the quality of the collected single-particle data was affected by beam-image shift, the optical properties for particles extracted from images recorded in individual positions in the 5 × 5 pattern were refined as independent optics groups. Thereafter, reconstructions were refined independently for the subsets of particles acquired at corresponding positions in the beam-image shift pattern (Table 3 ▸ and Supplementary Table S2). The number of extracted particles per position varied between 36 000 and 60 000, and the reconstructions reached very similar resolutions of between 1.97 and 2.07 Å, where the difference in resolution correlates with the number of particles in the data sets. Their sharpening and Rosenthal–Henderson *B* factors had only a narrow spread in the ranges 49–56 and 55–61 Å^2^, respectively (Table 3 ▸, Supplementary Table S2). This suggests that the quality of the data recorded with large beam-image shifts is homogeneous within a ±7 µm range and is direction-independent (Table 3 ▸, Supplementary Fig. S4). It is notable that the beam tilts refined for individual positions deviate from the average beam tilt by a maximum of 0.08 mrad. This indicates that the coma correction is accurate and stable over the period of data collection and is not a resolution-limiting factor at least to a resolution of 1.7 Å. No significant variations were observed for the higher-order aberrations, including trefoil and fourth-order aberrations, determined in relation to the data recorded at different positions of the 5 × 5 pattern (Supplementary Table S2). Indeed, refinement of the complete data set with 25 optical groups, and independent tilt refined for each group, improved the resolution by only 0.04 Å.

The average residual astigmatism in individual positions of the 5 × 5 pattern ranges from 140 to 870 Å (Table 3 ▸ and Supplementary Table S2; as expected from the measurements shown in Fig. 2 ▸ *d* and Supplementary Fig. S3*b*) and does not influence the quality of the single-particle reconstruction.

Cumulatively, this analysis indicates that coma can reliably be corrected on the CRYO ARM 300 for image-shift amplitudes of at least 7 µm and that the information in the images is preserved at least to a resolution of 1.7 Å.

## 4. Discussion   

Here, we characterized the performance of the CRYO ARM 300 electron microscope for single-particle data collection using coma- and astigmatism-corrected image shifts with amplitudes of up to 7 µm. This data-collection strategy allows the recording of multiple images from a single stage position, thus significantly increasing the throughput. We show that using the data-collection procedures established on our CRYO ARM 300, single-particle data can be routinely collected with throughputs of between 6000 and 9000 images per day. This is a more than 2.6-fold increase in data-collection throughput compared with previously reported data-collection rates (Fislage *et al.*, 2020 ▸).

This acceleration is due to a combination of factors. Although an increased number of images per stage position was the main focus of throughput improvement, a faster detector (K3 versus K2) that roughly quartered the exposure and image-recording time also contributed. A reduction of the time needed for stage alignment and the elimination of other unnecessary delays also played important roles. Analysis of the throughput clearly showed that one of the bottlenecks is associated with excessive time expenditure, needing an additional 4 s to set the beam-image shift, tilt and astigmatism of the electron beam. While this manuscript was under revision, JEOL updated the TEM Center from version 4.2.2 to 4.2.3, and the dead time associated with beam manipulation was reduced to approximately 0.6 s. As a result, data-collection throughput further increased to around 8000 images per day when using a data-collection strategy that acquired 20 images per stage position. This rate could be predicted using our model (Fig. 1 ▸, red curves), and it can now easily exceed 10 000 images per day. Our measurement shows that with a faster response time, the deflectors do not display any residual relaxation of image shift (Supplementary Fig. S5). Therefore, faster data collection should not compromise the quality of recorded micrographs.

We have characterized the optical properties of the microscope for beam-image shifts of up to 10 µm. We have also shown that the amplitude of beam-image shift-induced coma depends elliptically on the direction of the shift and increases linearly with the amplitude of the shift, with slopes along the principal elliptical axes of 0.19 and 0.31 mrad µm^−1^ (Supplementary Fig. S2*c*). These slopes are comparable to the values reported for Titan Krios microscopes (Thermo Fisher Scientific), although anisotropy of coma was not reported for Titan Krios (Cheng *et al.*, 2018 ▸; Wu *et al.*, 2019 ▸). The reasons for the azimuthal anisotropy of coma remain unknown and require further investigation. Here, we can only speculate that it may be caused by intrinsic properties of some of the optical elements or by inaccuracies in the adjustment of pivot points for image shift that result in beam tilt at the entrance to the in-column Ω energy filter. Compensation for aberrations, using a simple function that depends linearly on image shifts, is efficient in correcting coma and to a lesser degree astigmatism (Figs. 2 ▸ *b* and 2 ▸ *d*). The residual coma reveals a quasi-symmetric pattern, with its center of symmetry shifted off the coma-free center by a few micrometres.

The origin of such residual asymmetries is again not known. We believe that they may be caused by slight physical mis­alignment of condenser lenses or the in-column Ω energy filter.

Single-particle reconstruction of apoferritin with image shifts of up to 7 µm shows that coma compensation is efficient and accurate to within 0.1 mrad and that the residual astigmatism amplitude remains below 0.09 µm. No significant variation in higher-order aberrations associated with image shift can be detected at a resolution of 2 Å (Supplementary Table S3). Collectively, this suggests that coma-corrected single-particle data collection delivers data with very similar quality and optical properties over a range of beam-image shifts with amplitudes of at least 7 µm. This also suggests that quick data collection does not compromise the quality of the single-particle data, at least to a resolution of 1.7 Å.

The benchmark data set was collected with a single image acquired per hole. In actual practice, however, three images are recorded per 1.2 µm hole and five images are recorded per 2 µm hole. For the most commonly used Quantifoil grids with hole patterns of R 0.6/1, R 1.2/1.3 and R 2/1, the number of images recorded (per position with an amplitude of 7 µm) varies between 30 and 125. Thus, high-throughput collection can be achieved with all types of commonly used holey EM grids. It should be noted, however, that the choice of the pattern and the amplitude of the applied image shifts depend primarily on the availability of larger arrays of holes with homogeneous and optimal ice thickness.

With even larger image shifts of between 20 and 45 µm it becomes possible to collect images for a complete grid square from a single stage position for 400- and 200-mesh grids. In this mode, hundreds of images can be recorded from a single stage position, potentially increasing throughput by an additional 20–50%. Such an approach, however, requires more accurate coma and astigmatism correction, regular focusing and, even more importantly, the implementation of algorithms for the automated selection of optimal imaging areas. Consequently, procedures for applying beam-image shifts to variable patterns of holes for each selected square are also required.

An overnight data collection with a throughput exceeding 6000 images per day results in over 3000 images. For many cryo-EM samples, this represents a sufficient quantity of micrographs to produce a high-resolution reconstruction. At this data-collection rate, throughput is limited by the time needed to evaluate the quality of the cryo-EM grid, the collection of the grid atlas, the acquisition of square montages in *SerialEM* and the selection of holes with optimal ice thickness. These steps have already been automated in the *EPU* and *Leginon* software (Cheng *et al.*, 2021 ▸). Implementing similar automation within the framework of *SerialEM* would further significantly boost microscope throughput.

While collecting benchmark data, we did not aim to achieve the highest resolution, but rather to characterize microscope behavior for data collected with large image shifts. Reconstruction of the collected data set reached a resolution of 1.7 Å, which corresponded to a 0.87 fraction of the Nyquist resolution. The resolution here was improved compared with our previous benchmark because of the larger number of particles, as well as denser particle packing in the holes, enabling more accurate refinement of CTF parameters and particle polishing. An improvement in resolution was achieved despite the use of carbon, rather than UltrAuFoil, grids and the use of a larger pixel size (0.753 Å versus 0.603 Å). The Rosenthal–Henderson *B* factor of 68 Å^2^, which was calculated for the benchmark, is also comparable to the previously reported benchmark of 62 Å^2^ (Fislage *et al.*, 2020 ▸). It is also higher than the *B* factor of 47 Å^2^ reported for an apoferritin data set reconstituted to a resolution of 1.54 Å from data recorded on a similar microscope, albeit at higher magnification (Kato *et al.*, 2019 ▸). Multiple combinations of factors could account for the higher *B* factor, including the type of grid used (carbon instead of UltrAuFoil), which resulted in higher initial drift caused by local charging (Russo & Passmore, 2014 ▸). Suboptimal ice thickness could also be another factor out of many. As the resolution of the data set is close to the Nyquist limit, increasing the magnification may have further improved the resolution. Such improvement, however, would most likely have been moderate, because our data were collected in CDS mode on a K3 detector under near-optimal flux conditions, which have a high detective quantum efiiciency (DQE) up to the Nyquist resolution (Sun *et al.*, 2020 ▸).

Interestingly, similar to the previous benchmark, the distribution of points on the *B*-factor plot is clearly curved (Fig. 3 ▸ *d*), such that the *B* factor decreases with increased resolution. This makes it more difficult to increase the resolution beyond the current limit. The reasons for this may include limited stability of the beam tilt (with approximately ±0.1 mrad observed on our microscope), as discussed previously (Fislage *et al.*, 2020 ▸), and high average beam tilt. The latter may result in asymmetric aberrations that cannot be efficiently corrected beyond an achieved resolution. Combining a cFEG with an electron energy filter enables reconstruction at atomic resolution (Nakane *et al.*, 2020 ▸; Yip *et al.*, 2020 ▸). The factors causing the bending of the *B*-factor plot will need to be understood and eliminated in order to be able to take advantage of the full potential of the CRYO ARM 300 for high-resolution imaging.

To conclude, we have shown that the CRYO ARM 300 is suitable for high-throughput data collection using multiple exposures per stage position by applying coma-and-astigmatism-corrected beam-image shifts. The corrections are stable throughout the period of data collection. They are also accurate enough (for single-particle images acquired with beam-image shifts of up to at least 7 µm) to enable the 3D reconstruction of apoferritin to a resolution of 1.7 Å. The quality of single-particle data collected with such image shifts is indistinguishable from the quality of data collected on the coma-free axis. This enables a throughput exceeding 6000 images per day, and following the elimination of delays associated with setting beam-image shift, tilt and astigmatism, a throughput exceeding 10 000 images per day was achieved for routine, high-resolution data collection on this microscope.

The final 3D map and raw data were deposited in the EMDB and EMPIAR databases with access codes EMD-12358 and EMPIAR-10639.

## Supplementary Material

EMDB reference: apoferritin, EMD-12358

Supplementary Tables and Figures. DOI: 10.1107/S2059798321002151/id5009sup1.pdf

## Figures and Tables

**Table 1:** Timing of individual operations during single-particle data collection

Operation	Duration	Frequency	Symbol
Liquid-nitrogen dewar refilling	20 min	Every 13 h	*t*_LN_ †
Stage positioning	28 s	Per cycle	*t*_stage_
Focusing	26 s	Per cycle	*t*_focus_
Beam centering	6 s	Per cycle	*t*_beam_
Beam shift/tilt/stigmator	4 s	Per image	*t*_BS_
Stabilization delay	0 s	Per image	*t*_stab_
Image exposure and recording	3.3–5.6 s	Per image	*t*_rec_ ‡
*SerialEM* cycle overhead	0.15 s	Per image	*t*_SerialEM_
Ice-thickness measurement	5.5 s	Per cycle	*t*_ice_

† *t*_LN_ in (1) signifies the total average time spent in refilling liquid-nitrogen dewars and stage stabilization every 24 h.

‡ A range of times is shown for the shortest and longest exposure times used on our microscope.

**Table 2:** Data-collection parameters and statistics for the apoferritin data set

Data collection	
Electron microscope	CRYO ARM 300
Electron detector	K3, CDS mode
Voltage (kV)	300
Nominal magnification	60000
Exposure time (s)	3.37
Defocus range (µm)	0.3-1.5
No. of exposures per stage position	25
No. of frames	59
Pixel size (Å)	0.753
Electron dose (e^−^ Å^−2^)	60
No. of collected images	3125
3D reconstruction	
Final particles	702667
Applied symmetry	*O*
Resolution (Å)	1.71
Sharpening *B* factor (Å^2^)	−51
Rosenthal–Henderson *B* factor (Å^2^)	68

**Table 3:** Properties of apoferritin data acquired from individual positions of the 5 × 5 pattern averaged for positions with an identical amplitude of the beam-image shift

Shift radius (µm)	No. of images	No. of particles ×10^3^	Astigmatism amplitude (Å) ×100	FSC resolution (Å)	Sharpening *B* factor (Å^2^)	Rosenthal–Henderson *B* factor (Å^2^)	No. of averaged positions
0	112	57.5	1.85	1.97	50	59	1
2.5	112 ± 5	57 ± 3	2.1 ± 0.5	2.00 ± 0.01	52 ± 2	59 ± 2	4
3.5	112 ± 6	57 ± 4	2.7 ± 0.7	1.99 ± 0.02	51 ± 1	61 ± 4	4
5.0	104 ± 8	53 ± 5	3.7 ± 1.2	1.99 ± 0.01	51 ± 1	63 ± 3	4
5.5	103 ± 9	52 ± 5	4.3 ± 1.6	2.02 ± 0.03	51 ± 1	62 ± 3	8
7.0	98 ± 15	48 ± 8	6.3 ± 2.0	2.01 ± 0.04	51 ± 2	61 ± 3	4
