# Supplementary material for: Coma-corrected rapid single-particle cryo-EM data collection on the CRYO ARM 300
Source: Acta Crystallogr D Struct Biol. 2021 Apr 14;77(Pt 5):555–64. doi: 10.1107/S2059798321002151 (PMC8098478; doi:10.1107/S2059798321002151)
Supplement: Supplementary file 1 [file d-77-00555-sup1.pdf]

STRUCTURAL
BIOLOGY

Volume 77 (2021)

Supporting information for article:

Coma-corrected rapid single-particle cryo-EM data collection on the cryoARM300

Rouslan G. Efremov and Annelore Stroobants

Table S1 The time required to acquire an image on K3 detector in SerialEM for a range of exposure times per frame, number of frames and active or inactive CDS mode.

CDS active	Exp time per frame (s)	Number of frames	Exposure time (s)	Total time (s)
yes	0.057	30	1.72	3.59
yes	0.057	59	3.38	5.59
yes	0.057	120	6.87	8.74
yes	0.1	30	3.00	4.86
yes	0.1995	30	5.99	7.86
no	0.0355	30	1.06	3.32
no	0.0355	60	2.12	4.70
no	0.0575	30	1.72	3.55
no	0.0575	59	3.38	5.20
no	0.0575	120	6.87	8.68

Table S2 Properties of datasets collected from individual positions of the 5x5 pattern and refined independently from each other.

position	IS radius (um)	IS angle	Nr of images	Nr of particles	Astigmatism amplitude (Å)	Resolution (Å)	Sharpening B-factor (Å²)	Beam tilt X (mrad)	Beam tilt Y (mrad)	Beam tilt amp (mrad_	Rosenthal-Henderson B-factor (Å2)	Symmetrical Zernike polynomial coefficients								Antisymmetrical Zernike polynomial coefficients				
1	7.0	358	77	36,519	874	2.07	-50	-0.35	-0.86	0.93	63	4.18	1.35	7.60	4.55	1.63	0.60	3.46	1.69	1.17	-0.49	-0.29	0.10	-0.89
2	5.6	17	93	45,853	410	2.01	-51	-0.33	-0.88	0.94	58	4.23	0.75	7.69	5.13	1.29	0.39	3.51	1.88	0.75	-0.54	-0.24	0.09	-0.12
3	5.0	43	93	45,984	229	1.99	-49	-0.30	-0.89	0.94	61	4.21	1.04	7.66	6.42	1.02	0.50	3.50	2.36	0.46	-0.60	-0.15	0.40	-0.29
4	5.6	69	97	47,795	271	2.05	-50	-0.28	-0.91	0.95	61	4.49	0.86	8.09	6.79	0.63	0.43	3.66	2.49	0.59	-0.64	-0.09	1.21	-0.79
5	7.0	88	96	46,114	399	1.97	-51	-0.27	-0.90	0.94	58	4.57	1.29	8.22	7.65	0.35	0.58	3.70	2.81	0.22	-0.63	-0.04	2.29	-1.18
6	5.6	106	103	52,018	336	1.99	-52	-0.25	-0.90	0.94	65	4.83	0.70	8.64	7.44	0.56	0.37	3.85	2.74	0.22	-0.63	0.00	3.16	-0.58
7	3.5	88	110	55,447	189	2.01	-52	-0.27	-0.88	0.92	66	4.56	1.08	8.20	7.67	0.90	0.51	3.69	2.82	0.31	-0.56	-0.05	1.97	-0.20
8	2.5	43	107	54,149	143	1.99	-56	-0.29	-0.86	0.91	56	4.38	1.04	7.93	6.97	0.90	0.49	3.59	2.56	0.15	-0.49	-0.12	1.11	0.10
9	3.5	358	104	51,651	333	2.01	-51	-0.31	-0.83	0.89	62	4.22	1.35	7.67	6.21	1.27	0.61	3.50	2.29	0.58	-0.42	-0.19	0.82	-0.03
10	5.6	340	91	44,610	717	2.05	-53	-0.33	-0.82	0.89	58	4.06	1.31	7.41	6.20	1.54	0.59	3.40	2.28	1.00	-0.39	-0.24	0.46	-0.50
11	5.0	313	101	51,290	597	2.01	-53	-0.31	-0.81	0.87	67	4.34	1.02	7.86	6.50	1.77	0.49	3.56	2.39	0.22	-0.35	-0.17	0.51	-0.24
12	2.5	313	112	56,991	273	2.01	-51	-0.29	-0.82	0.87	58	4.36	0.95	7.89	6.79	1.42	0.47	3.58	2.50	-0.04	-0.38	-0.11	0.93	0.20
13	0.0	0	112	57,533	185	1.97	-50	-0.26	-0.84	0.88	59	4.58	1.07	8.25	7.37	0.58	0.51	3.71	2.72	-0.16	-0.44	-0.04	1.63	0.46
14	2.5	133	111	56,997	221	2.01	-52	-0.25	-0.85	0.89	60	4.79	0.50	8.58	7.41	0.60	0.30	3.83	2.73	-0.10	-0.49	0.02	2.58	0.44
15	5.0	133	110	55,832	412	1.99	-52	-0.23	-0.86	0.89	60	5.02	0.30	8.93	8.13	0.04	0.23	3.95	2.99	0.00	-0.51	0.07	3.84	0.24
16	5.6	160	110	55,483	471	2.05	-51	-0.22	-0.85	0.88	65	4.99	0.08	8.88	8.07	0.13	0.15	3.94	2.97	-0.32	-0.47	0.09	4.52	1.01
17	3.5	178	116	58,865	219	1.99	-50	-0.24	-0.83	0.87	61	5.02	0.48	8.92	7.89	0.35	0.30	3.95	2.90	-0.05	-0.43	0.04	3.21	1.01
18	2.5	223	118	60,948	222	1.99	-51	-0.25	-0.81	0.85	60	4.85	0.88	8.67	7.33	0.80	0.44	3.87	2.70	-0.33	-0.37	0.00	2.06	1.09
19	3.5	268	116	60,830	328	1.97	-51	-0.27	-0.79	0.84	55	4.56	0.95	8.21	7.52	1.10	0.47	3.70	2.77	-0.37	-0.30	-0.06	0.93	0.79
20	5.6	286	109	55,340	553	1.99	-50	-0.29	-0.78	0.83	65	4.52	1.88	8.16	6.78	1.45	0.81	3.68	2.50	-0.30	-0.25	-0.11	0.15	0.19
21	7.0	268	110	54,944	673	1.99	-50	-0.26	-0.78	0.83	59	4.59	1.33	8.26	6.74	1.18	0.61	3.72	2.48	-0.79	-0.28	-0.05	-0.12	1.19
22	5.6	249	115	57,795	437	1.99	-51	-0.25	-0.79	0.83	59	4.60	1.39	8.28	7.12	0.86	0.62	3.73	2.62	-0.55	-0.30	0.00	1.05	1.72
23	5.0	223	111	57,200	243	1.99	-51	-0.23	-0.80	0.84	62	4.86	0.80	8.69	7.91	0.47	0.41	3.88	2.91	-0.46	-0.34	0.06	2.36	1.87
24	5.6	197	109	53,836	218	2.01	-51	-0.22	-0.81	0.84	62	5.18	0.10	9.19	8.16	0.00	0.15	4.05	3.00	-0.15	-0.36	0.11	3.80	1.87
25	7.0	178	108	52,608	575	2.01	-54	-0.21	-0.82	0.85	65	5.31	0.30	9.38	8.02	0.00	0.22	4.12	2.95	-0.68	-0.40	0.12	5.12	1.85

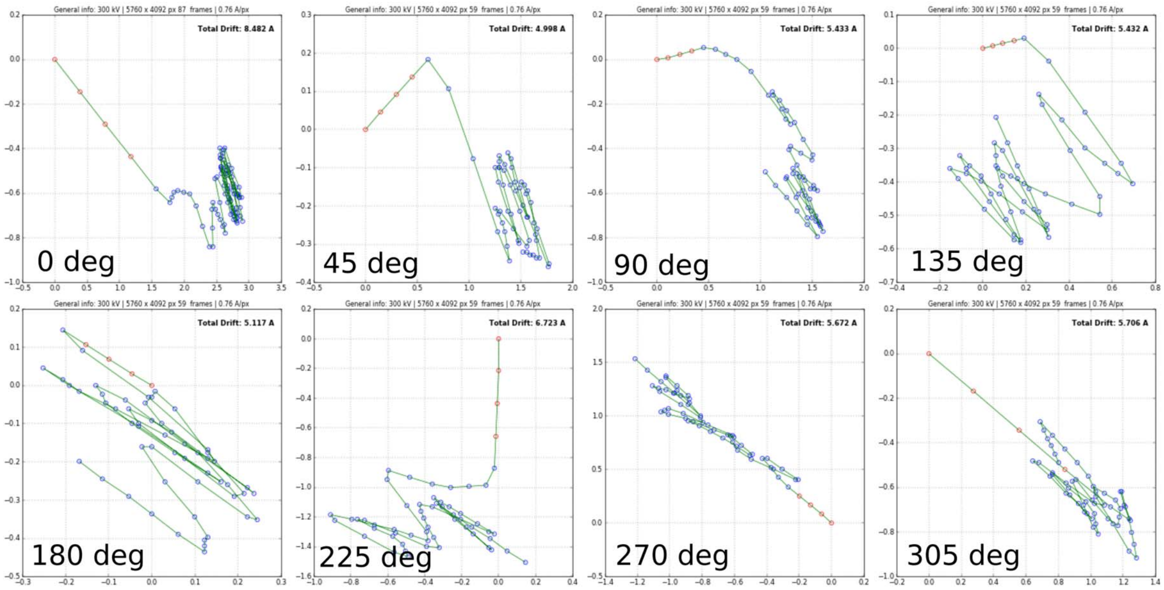

Figure S1 Measurement of image shift induced drift. The traces are shown for images recorded with per frame exposure of 0.057s and a total of 87 frames. The frame recording was initiated immediately after completion of coma and astigmatism corrected beam-image shift from neutral position to a position at indicated angle and amplitude of 10 um. The angle indicated on the panel is measured relative to IS_X deflector axis. The frames corresponding to early exposure are marked with red circles.

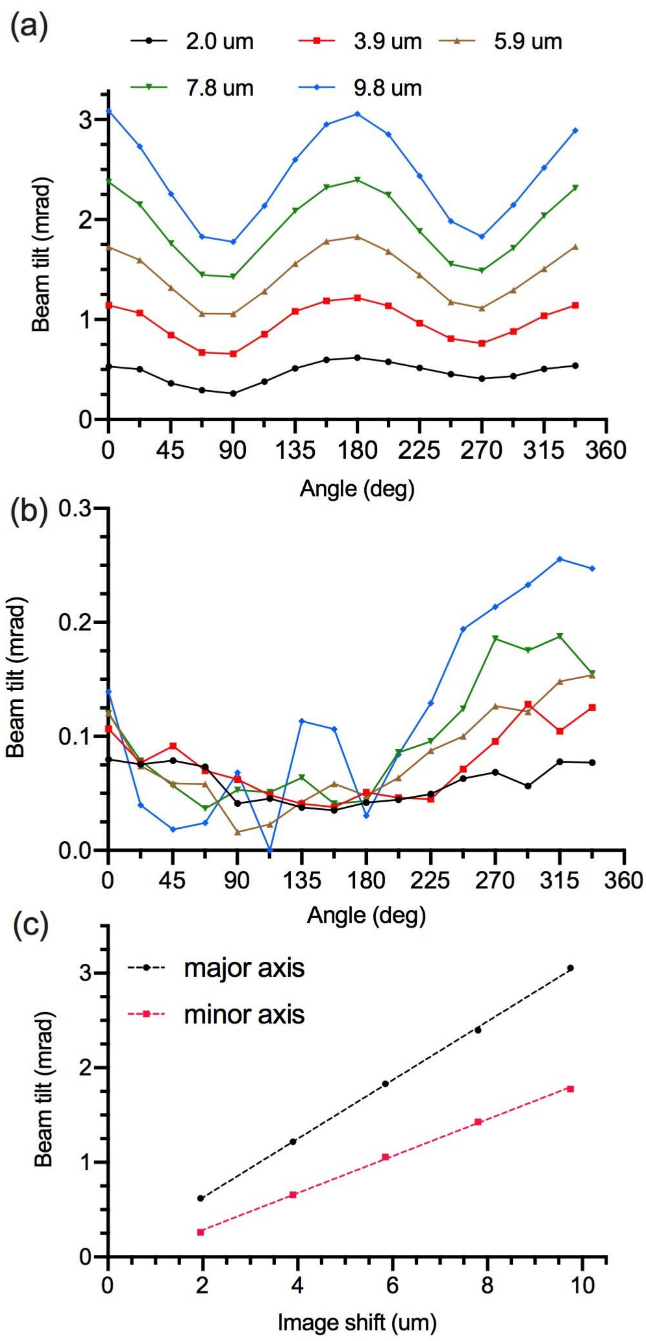

Figure S2 Amplitudes of beam-image shift induced coma before (a) and after (b) applying correction. Amplitude of coma is shown as a function of image shift angle for equidistant image shifts. (c) Liner fit of the coma amplitude induced along principal directions of the ellipse. The amplitude of tilt for the linear fit has values of 0.19 mrad/um and 0.31 mrad/um for minor and major ellipsoidal axis, respectively.

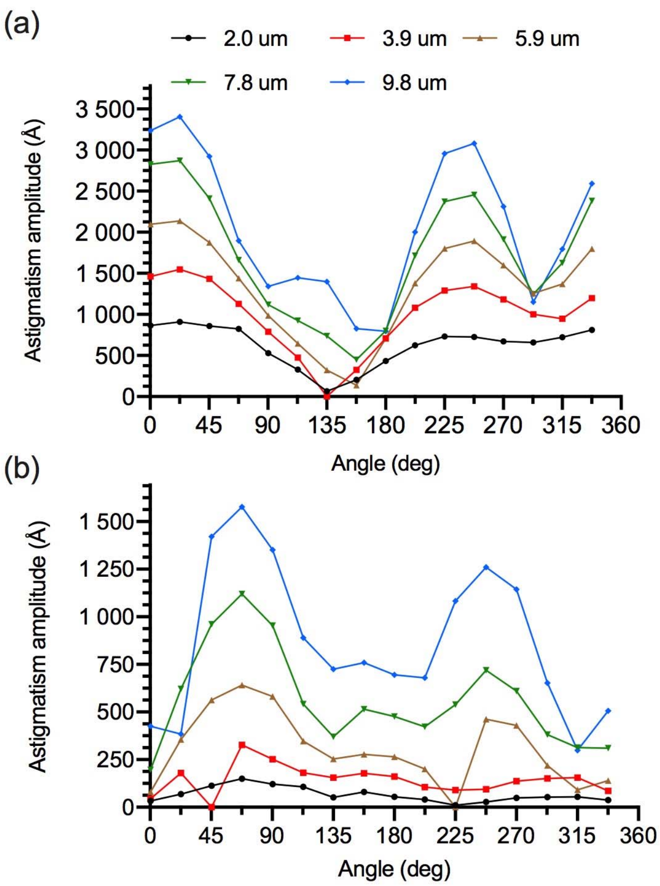

Figure S3 Beam-image shift induced astigmatism before (a) and after (b) applying correction. Amplitude of astigmatism is shown as a function of image shift angle for equidistant beam-image shifts.

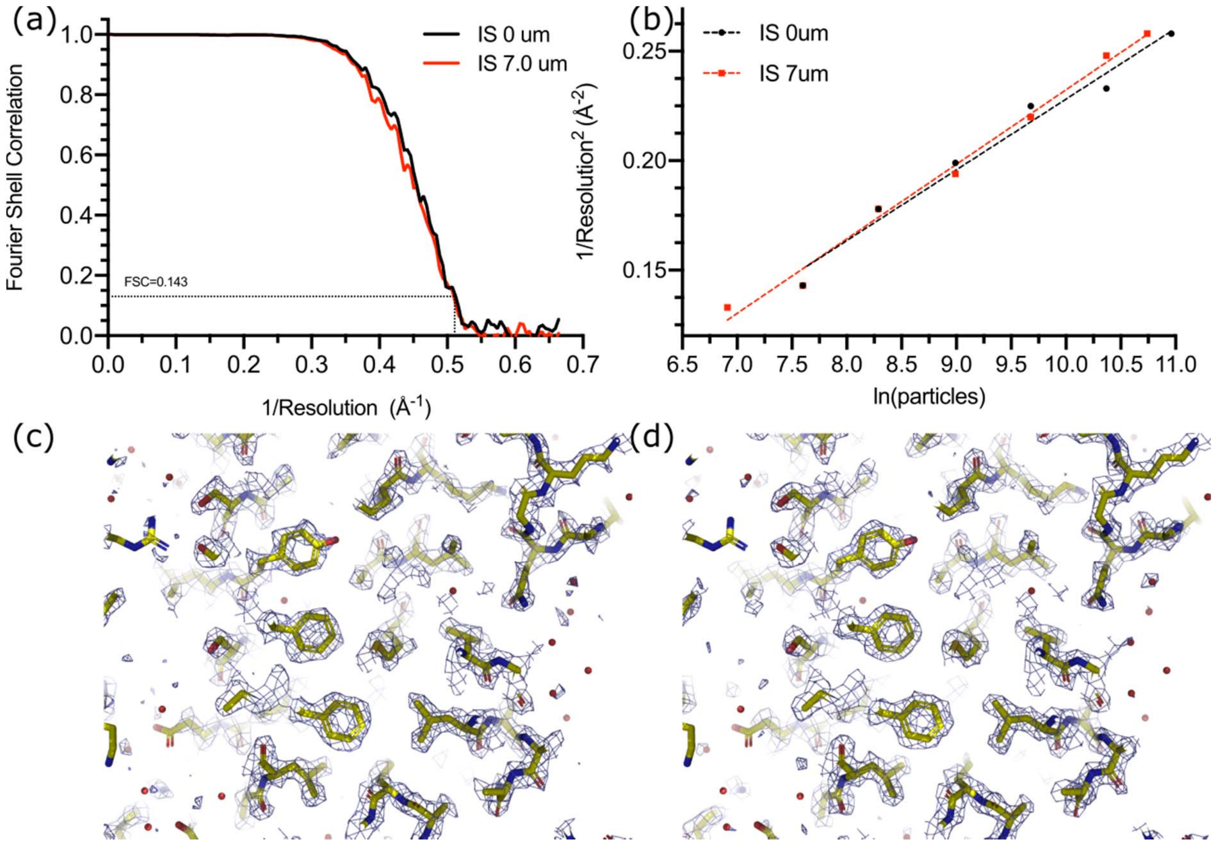

Figure S4 Comparison of the properties of data recorded on the coma-free axis and with 7 um beam-image shift and aberration correction. (a) Fourier Shell Correlations for masked densities, (b) Rosenthal-Henderson B-factor plots. Densities of the reconstructions calculated from data recorded on coma-free axis (c) and with 7 um beam-image shift (d).

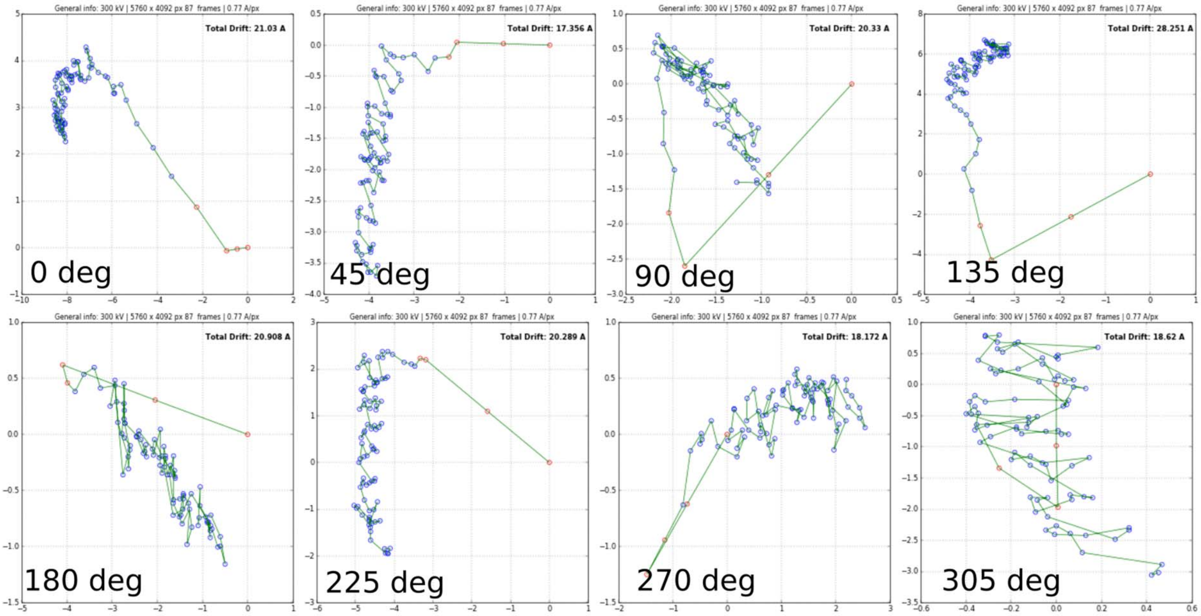

Figure S5 Measurement of beam-image shift induced drift with faster response time of deflectors. The measurements were performed under conditions identical to those of Figure S1 but using version of TEM Center 4.2.3 in which time required to change deflectors setting was reduced to 0.6 s as compared to version 4.2.2 in which the process took 4 s and for which data are shown in Figure S1.
